# Sustainable Seawater Desalination and Energy Management: Mechanisms, Strategies, and the Way Forward

**DOI:** 10.34133/research.0290

**Published:** 2023-12-20

**Authors:** Meng Wang, Yen Wei, Ruoxin Li, Xin Wang, Chengyu Wang, Nanqi Ren, Shih-Hsin Ho

**Affiliations:** ^1^ State Key Laboratory of Urban Water Resource and Environment, School of Environment, Harbin Institute of Technology, Harbin 150001, China.; ^2^ Department of Chemistry, Tsinghua University, Beijing 100084, China.; ^3^ Key Laboratory of Bio-based Material Science & Technology (Northeast Forestry University), Ministry of Education, Harbin 150040, China.

## Abstract

Solar-driven desalination systems have been recognized as an effective technology to address the water crisis. Recently, evaporators prepared based on advanced manufacturing technologies have emerged as a promising tool in enhancing ocean energy utilization. In this review, we discussed the thermal conversion, energy flow, salt deposition mechanisms, and design strategies for solar-driven desalination systems, and explored how to improve the desalination performance and energy use efficiency of the systems through advanced manufacturing technologies. In future perspectives, we determined the feasibility of coupling solar-driven solar desalination systems with multi-stage energy utilization systems and emerging artificial intelligence technologies, for which conclusions are given and new directions for future desalination system development are envisioned. Finally, exciting opportunities and challenges in the face of basic research and practical implementation are discussed, providing promising solutions and blueprints for green and novel desalination technologies while achieving sustainable development.

## Introduction

Water scarcity largely threatens human survival and social development [1–3]. It is estimated that more than half of the global population will face clean water shortage by 2050 as population growth and pollution worsen [4,5]. Ensuring access to reliable and safe drinking water for all is a global challenge and is formally recognized as an international development priority by 2030 in the UN Global Development Priorities Framework (SDG 6.118) [6]. The growing increase in demand for potable water is considered to be the main driver of the global desalination market, and as a result, there is a growing global interest in solutions that provide safe drinking water without contributing to large carbon emission associated with the growing dependence on fossil fuel, while reducing the negative impact on the global environment [7–9]. It is well known that the total water resources on Earth are certain; the salty seawater that cannot be directly consumed accounts for 97.5% of the total water resources [10]. Thus, the efficient conversion of seawater into freshwater is an important issue for researchers working to address the sustainable development of human society.

The process of seawater desalination requires additional energy consumption and separation technology [11,12]. Recent studies on freshwater generation have shown that technologies such as distillation (including multi-stage flash distillation, pressure vapor distillation, and multi-effect evaporation), membrane separation, freezing, and electrodialysis require high consumption of fossil fuel, substantial investment, and greenhouse gas emission and are therefore not the best solution to meet sustainable development [13–16]. To do this, solar-driven systems for sustainable seawater desalination are recommended as excellent candidates for seawater purification and wastewater treatment due to their cost-effectiveness, environmental friendliness, and sustainability [17–19]. Based on the above, researchers have proposed an interfacial solar vapor generation (SVG) system that couples solar-driven photothermal heat-to-vapor conversion and localizes it at the seawater evaporation interface, thereby improving the vapor generation efficiency [20–23]. However, the desalination system based on the interfacial evaporation effect usually encounters the problems that cannot be ignored during the long-term operation—salt accumulation and poor energy management [24,25]. The bulk seawater is transported to the top of the insulation due to the drive of capillary force, while the photothermal material causes the upstream seawater in that part to evaporate violently [26–28]. Due to the unbalanced water supply rate and evaporation rate, the salt in seawater at the evaporation interface would be saturated and precipitated after long-term operation, which would not only reduce the actual light receiving area, but also block part of the transmission channel and even destroy the photothermal conversion layer, leading the evaporation system unreliable [29,30]. Meanwhile, the photothermal material in the desalination system is usually quite sensitive to the optical power density, which makes it difficult to maintain an efficient evaporation rate when the solar irradiation is insufficient [31–33]. Previous studies have often neglected the energy loss caused by heat leakage from a solar-driven photothermal evaporator to the surrounding environment, focusing on a single thermal energy utilization approach such as water vapor that often fails to achieve optimal energy utilization, thus resulting in low energy management efficiency.

Among the possible solutions, solar desalination systems fabricated based on advanced manufacturing technologies are the most competitive due to their high photothermal conversion efficiency, high structural tunability, and excellent salt deposition resistance [34]. Solar-driven interfacial desalination systems constructed by 3D printing, bionic technologies, and microfluidics that provide membranes or components with controlled characteristics and morphology can possess an optimized water channel structure (enhanced salt reflux) and a wettability conversion interface to prevent salt accumulation and localize salt precipitation structure, effectively avoiding damage to the system from salt accumulation while improving evaporation efficiency [35–38]. It is encouraging that new photothermal nanomaterials such as graphene [39,40], carbon nanotubes (CNTs) [41,42], plasma metals [43–45], and semiconductors [46] could be easily applied to current advanced manufacturing technologies to enhance solar energy absorption and thus increase the energy density of water evaporation. From the perspective of energy management, the solar-driven desalination system prepared based on advanced manufacturing technology has excellent intersection with energy storage, thermal cycle, and energy multi-stage utilization due to high desalination efficiency and unique controllable structure, such as coupling with phase change energy storage [47,48], salinity gradient energy [49], tidal energy [50], and triboelectric nanogenerators [51]. Meanwhile, emerging machine learning (ML) algorithms have also been introduced as powerful tools for predicting the performance and productivity of desalination systems in order to optimize the long-term energy management [52,53]. However, although some reported reviews have emphasized the performance, manufacturing technology, and applications of nanomaterial-based solar-driven interfacial evaporation systems, a comprehensive review dealing with solutions for salt accumulation and energy management (multi-stage energy utilization and artificial intelligence [AI] prediction) in desalination systems is still limited.

The aim of this review is to comprehensively analyze the solar-driven desalination systems for combat salt deposition and energy management, identify critical gaps in knowledge, and summarize mechanisms and strategies based on advanced manufacturing technologies to improve the performance and salt prevention of desalination systems. Then, we propose to use the concept of multi-system coupling with AI to improve the energy management efficiency of desalination systems. The main focus involves the following: (a) discussing the thermal conversion, energy flow, and salt deposition mechanisms of solar-driven desalination system; (b) evaluating the performance and efficiency of different desalination systems based on advanced manufacturing techniques; (c) examining design strategies for salt deposition resistance in desalination systems prepared by advanced manufacturing techniques; (d) determining the feasibility of coupling solar-driven solar desalination systems with multi-stage energy utilization systems and emerging AI technologies; and (e) highlighting the remaining challenges and envisioning the new directions for future desalination system development. This can help unlock the full potential of solar desalination systems and provide a sustainable route to address the current water crisis.

## Mechanisms of Salt Deposition during Desalination

### Basic concept of interfacial effect for solar desalination

Solar-driven desalination systems begin by converting solar radiation into thermal energy, which is then utilized to generate water vapor for producing clean water [54]. The entire interfacial evaporation process is predicated on the need for the material to absorb the incident solar flux efficiently and then convert it into thermal energy. High-performance photothermal materials are the basis for desalination utilizing solar energy [55,56]. Since the energy driving the interfacial evaporation effect originates from solar energy, the light absorption capacity of the material has a definite dependence on the evaporation rate. The absorption capacity for solar light is assessed by Eq. 1:

$$Q_{\text{solar}}=c_{\mathrm{opt}}\times I\times A_{\text{proj}}\times \alpha$$ (1)

where *c_opt_*, *I*, *A_proj_*, and *α* are the optical concentration, direct solar illumination intensity, irradiation area, and absorption coefficient, respectively. The ideal light-absorbing layer is free of transmission and reflection while converting visible light to heat energy with maximum efficiency. Therefore, increasing *α* can enhance the solar energy utilization efficiency of evaporation system. Three mechanisms for high light absorption have been proposed: (a) preparation of multilayer structures to meet anti-reflection requirements, (ii) high-density optical modes of photonic crystals, and (iii) coupling with light-absorbing nanostructures for efficient utilization of light energy [8,57–60]. The solar absorber converts sunlight into infrared radiation (IR). Since the penetration depth of IR in water is about 10 μm, most of the heat radiation can be absorbed in the thin water layer to realize interfacial evaporation [61,62]. The configuration design of interfacial evaporation is divided into 2 types under solar irradiation, the surface of the photothermal material generates local heat sites by absorbing solar energy, and seawater is heated to generate water vapor by flowing capillary through the fine channel structure of the photothermal absorber to these heat sites. The other is the employment of a secondary path structure to transport seawater, which is an insulation with complex pore structure that separates the photothermal zone from the bulk seawater to improve the efficiency of thermal localization. The second interfacial evaporation design achieves 4 main functions: (a) sunlight is captured and converted into heat, (b) evaporation of water through fixed domain heating, (c) passive water supply using capillary forces for continuous evaporation, and (d) isolation from bulk water to reduce heat transfer.

### Evaporation rate and energy efficiency calculation

For interfacial evaporation desalination systems, conversion of sunlight to high-temperature steam is the most important energy flow process, and the accurate expression of sunlight–vapor conversion efficiency a critical metric for assessing the system efficiency. The evaporation rate (*v_e_*) is defined as the solar-driven vapor production rate per unit time and unit area under steady-state conditions, which can be described by Eq. 2:

$$v_{e}=\frac{\mathrm{dm}}{A_{\text{proj}}\times \mathrm{dt}}$$ (2)

where *m* and *t* represent the mass change and time, respectively. In most cases, the interfacial evaporator needs to operate for a certain time period before it reaches a steady state, and it is accurate to use a linear fit of the slope of the mass change at steady state to perform the calculation. The energy utilization of this process can be evaluated in terms of solar energy conversion efficiency:

$$\eta =\frac{\overset{\cdot }{m}\left(C_{p}\times \Delta T+\Delta H_{\mathrm{vap}}\right)}{Q_{\text{solar}}}$$ (3)

where $\overset{\cdot }{m}$, *C_p_*, and *ΔH_vap_* are the evaporation rate, the specific heat capacity of water (4.18 J g^−1^ K^−1^), and the latent heat of evaporation (2,257 kJ kg^−1^), respectively. The key elements of an efficient solar-driven interfacial vapor generation system include efficient solar energy absorption and heat-to-vapor conversion, but all current systems incur energy losses (heat transfer into the bulk seawater and convective heat transfer with the surrounding air, as well as thermal radiation to the surrounding environment) that prevent high levels of input solar energy utilization.

### Mechanism of salt deposition during desalination

Apart from the optimal design of system structures, salt deposition under continuous operating conditions is a serious obstacle to maintaining and improving the performance of solar desalination systems, and this is one of the major issues that the technology now needs to address [63].

**Fig. 1.**
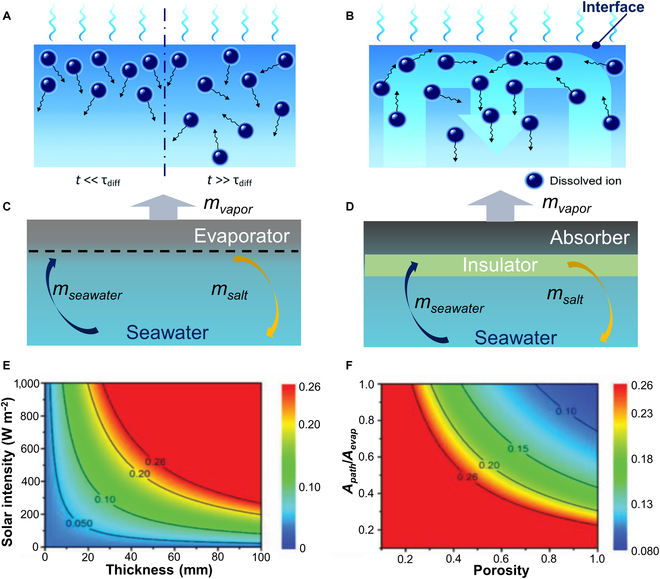
(A and B) Salt diffusion reaches steady state when *t* > *τ*_diff_; schematic shows salt convection due to concentration and temperature-induced density gradient. Reproduced from [18] with permission from the Royal Society of Chemistry. (C and D) Schematics of salt and water transport in the interfacial solar evaporation system without and with water supply path management. (E and F) The maximum salt concentration (at the evaporation surface) for different solar intensity and thickness of absorber and the maximum salt concentration for different porosity of wick and area ratio *A_p_*/*A_e_*. Reproduced from [64] with permission from Wiley-VCH.

Salt deposition is a common concomitant of the interfacial solar desalination process. Evaporation efficiency and convective diffusive transport of salt ions jointly determine the salt concentration on the evaporation interface. During continuous evaporation, the salinity gradient between the evaporation interface and the bulk seawater increases, and diffusion forces will drive the migration of salt ions from the evaporating surface into the underlying bulk seawater [21]. The transport of salt ions in seawater is governed by the convection–diffusion equation:

$$\frac{\partial C}{\partial t}=D_{s,\mathrm{eff}}\nabla^{2}c-\nabla \cdot \left(\mathrm{uc}\right)$$ (4)

where *t*, *D*_*s*, *eff*_, and *u* represent time, the effective mass diffusivity of salt ions in the evaporator, and the flow field, respectively. The *D*_*s*, *eff*_ of an interfacial evaporator with a homogeneous porous structure can be reasonably estimated according to Eq. 5:

$$D_{s,\mathrm{eff}}=E_{P}^{\frac{3}{2}}D_{S}$$ (5)

where $E_{P}$ and *D_S_* are the porosity and the intrinsic mass diffusivity of salt ions in water, respectively. The evaporation system generates an interfacial water evaporation effect during operation while dissolved salt ions continue to remain in the unevaporated interfacial water, which leads to the formation of a salinity gradient along the capillary flow direction of the evaporator. The high concentration of salt ions is driven by the salinity gradient to diffuse from the evaporation interface into the low concentration of bulk seawater; that is, the interface water salinity of the evaporator is always maintained at a high level (>3.5 wt.%). Furthermore, due to the low mass diffusivity (*D_S_*) of salt ions in water (~10^−9^ m^2^ s^−1^), the characteristic time scale of salt diffusion is:

$$\tau_{\text{diff}}=\frac{L_{c}^{2}}{D_{S}}$$ (6)

where *L_c_* is the thickness of the solar evaporator for 1D diffusion of salt ions. Therefore, *τ_diff_* requires a certain amount of time (possibly several hours) to reach the equilibrium (Fig. 1A and B).

From Eqs. 4 to 6, it can be concluded that whether the salt crystals nucleate depends on the dynamic balance between the convection of ions within the capillary structure and the spontaneous diffusion of salt subjected to the salinity gradient. Since the gradient diffusion of salt ions requires a sufficiently long characteristic time scale, the salt is not able to be saturated (26 wt.%) when the gradient diffusion rate is greater than or equal to the salinity increase rate generated by capillary forces, and conversely, the salt can gradually reach saturation and nucleates at the evaporation interface. Under a determined evaporation rate (*v_e_*), the reasonable optimization of evaporator structure design to regulate the ions transport can avoid the influence of salt deposition (Fig. 1C and D). In steady state, the increase in concentration at the evaporation interface is equal to the decrease in concentration caused by diffusion:

$$\frac{v_{e}C_{b}A_{p}}{\left(1-C_{b}\right)A_{e}}=\frac{D_{\text{NaCl}}\rho E_{P}\left(C_{e}-C_{b}\right)}{L}$$ (7)

where *C_b_*, *C_e_*, *D_NaCl_*, *ρ*, *L*, *A_p_*, and *A_e_* represent the mass fraction of salt in bulk seawater, the mass fraction of salt at the evaporation interface, the mass diffusion coefficient of NaCl in seawater (~2×10^−9^ m^2^ s^−1^), the density of seawater, the thickness of seawater evaporator, the area of the evaporation interface, and the area of the capillary structure wick, respectively. Based on Eq. 7, Liu et al. [64] calculated the concentration on evaporated surfaces for various solar intensities and evaporator thicknesses. Combining Eqs. 2 and 3, in the case of *A_p_*/*A_e_* = 1, the solution at the interface becomes saturated and precipitates when the overall thickness of the evaporator is greater than 2.5 cm under the irradiation of AM1.5G. If *A_p_*/*A_e_* < 0.4, even ordinary porous evaporators of small *L* (~1 cm) are capable of generating salt precipitation (Fig. 1E and F).

From the above, we can see that whether the evaporation interface produced salt precipitation is closely related to light intensity, evaporation area, porosity, capillary structure wick, and thickness. Practically, the evaporator should be optimized under the light intensity of AM1.5G, and the internal porosity, thickness, and capillary structure core of the evaporator with uniform structure should be reasonably regulated to prevent salt precipitation. The anti-salt accumulation strategies based on the above theories are mainly divided into the following: (a) reasonable regulation of the *A_p_*/*A_e_* ratio and the design of a structured self-driven dissolved salt strategy utilizing salinity gradient to quickly transport salt to bulk seawater, (b) leveraging evaporator wettability switching interface to isolate salt from the photothermal structure, leading to a spontaneous generation of salinity gradient existed in bulk seawater, and (c) through a subtle structural design, inducing salt to be precipitated in specific areas of the evaporator in a targeted manner.

### Flow mechanism and energy management strategies

In a solar desalination system, light energy is first converted into thermal energy, and the thermal energy is transferred to the water–gas interface to produce hot vapor through heat conduction of the material or structure [65]. The energy loss from the multi-level transfer of energy affects the overall evaporation efficiency. To achieve high thermal efficiency, heat loss due to conduction, convection, and radiation should be minimized to maximize the effectiveness of heat utilization for steam generation [66,67]. These heat conversion and transfer characteristics demonstrate the importance of energy management for an evaporation system.

The following energy flows are included in the solar-vapor conversion process: (a) the heat consumed by evaporation from the interface water, (b) heat transfer to the bulk water, (c) convective heat transfer with surrounding air, and (d) thermal radiation to the environment. The energy flows of the system in equilibrium operation can be described as:

Aprojαqsolar=CbmΔTb+AprojhTe−T0+AprojκΔTeΔT0+ϵσAprojTe4−T04 (8)

where *q_solar_*, *C_b_*, *m*, *ΔT_b_*, *h*, *T_e_*, *T*_0_, *κ*, $\frac{{\Delta T}_{e}}{{\Delta T}_{0}}$, *ϵ*, and *σ* are incident light density, the specific heat capacity of bulk water, the mass of bulk water, the temperature change of bulk water, the heat transfer coefficient of natural convection, evaporation interface temperature, ambient temperature (~27 °C), the heat transfer coefficient of evaporator, the temperature gradient between evaporator and ambient, the thermal emissivity of evaporator, and the Stefan–Boltzmann constant (5.67 × 10^−8^ W m^−2^ K^−4^), respectively. From the above, it follows that the higher evaporation efficiency is not attained because the absorbed solar energy is wasted in conduction, radiation, and convection. For example, if no thermal or energy management is designed for the photothermal evaporation materials, the energy is estimated to be lost in radiation by 7%, convection by 5%, and conduction by 43%. The main energy management strategy for solar desalination systems is to reduce the heat transfer *κ* from the evaporator, such as using insulation made of porous materials, which can minimize heat transfer loss to the bulk water effectively. Expanding the effective evaporation area allows more vapor to escape and then consume more enthalpy of phase transformation (Δ*H*), thus suppressing the heat radiation loss of the system. Meanwhile, adding insulation to the exterior of the evaporator to reduce the radiation heat transfer (reducing $T_{e}^{4}-T_{0}^{4}$) is also an effective means of improving heat utilization.

The above strategies focus on endogenous energy efficiency improvements that are intrinsically improving the vapor production rate and energy utilization. In practice, intermittent solar energy, latent heat storage, utilization of hot steam, and utilization of ocean energy are often neglected, and these aspects, although limited by the efficiency and stability of desalination systems, open new avenues for improving the multi-level utilization of different energy. The energy multi-level utilization system coupled with interfacial desalination unit requires subtle structural design and reasonable energy flow configuration, and the optimization of system design and energy efficiency prediction leveraging advanced manufacturing technology and AI lay a solid foundation for the realization of the coupled system.

## Solar Desalination System Based on Advanced Manufacturing Technology

Increasing research interest in alleviating water scarcity has led to innovative design and optimization of interfacial evaporation desalination systems [68]. Emerging desalination systems based on resistance to salt deposition and energy management rely on structural design, energy flow optimization, and coupling with multiple systems, which place severe demands on the corresponding fabrication technologies and methods [69,70]. New desalination systems fabricated based on advanced manufacturing technologies may meet these requirements, from optimization of interface structures to producing 3D devices with complex geometries. This rapidly emerging technology offers the combined advantages of flexibility in structural design and material utilization, while reducing production cycle time and raw material waste.

### 3D printing

Based on the basic understanding of salt transport in the “Mechanism of salt deposition during desalination” section, the design of structured self-driven soluble salts by 3D printing with reasonable regulation of the *A_p_*/*A_e_* ratio is proved to be effective. 3D printing involves the layer-by-layer deposition of materials through the combined motion of the print head and print bed in the *X*, *Y*, and *Z* directions, providing the satisfaction of being able to precisely control the desired eigenform to achieve excellent evaporation efficiency and salt resistance, with excellent mechanical and chemical stability.

**Fig. 2.**
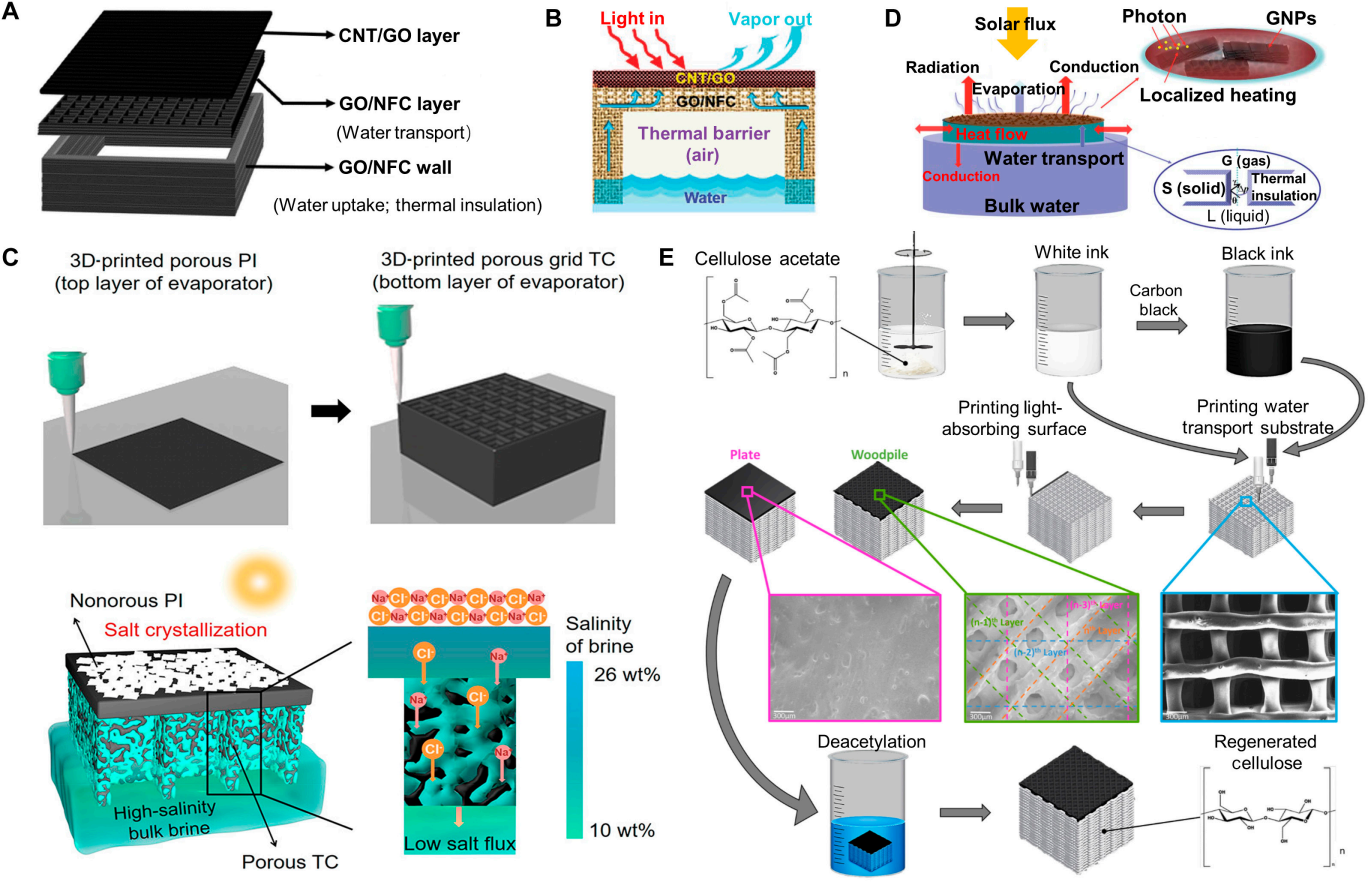
Solar desalination system based on advanced manufacturing technology. (A and B) Schematic illustration of the structure of the 3D-printed evaporator and solar steam generation of the 3D-CG/GN. Reproduced from [5] with permission from Wiley-VCH. (C) The fabrication process of 3D printing of a hierarchical porous evaporator and the low salt transport flux-induced salt crystallization on an evaporator with nonporous PI and porous TC. Reproduced from [71] with permission from the American Chemical Society. (D) Schematic of the floating structure of the 3D-printed interfacial solar steam generator with capillary water channels and a graphene coating. Reproduced from [72] with permission from the Royal Society of Chemistry. (E) Schematic of the fabrication process of the 3D-printed highly amorphous regenerated cellulose-based solar evaporator. Reproduced from [73] with permission from Elsevier.

A concave structured all-in-one evaporator with high porosity and solar absorption was constructed by Li et al. through 3D printing (Fig. 2A). The porous hydrophilic graphene oxide/nanofibrillated cellulose (GO/NFC) wall was acted as a support to form a continuous bottom-up water transport channel, which can largely enhance the salinity diffusion rate to inhibit interfacial salt deposition (Fig. 2B). According to Eq. 5, the extremely high structural porosity ($E_{P}$) effectively improves *D*_*s*, *eff*_ (effective mass diffusivity of salt ions), and the excellent salt suppression strategy can stabilize the system output for 50 cycles [5]. Improvements in desalination performance of highly concentrated brine can be achieved by engineering diffusion/convection resistance designs. He et al. demonstrated a strategy to tailor the directional salt transport flux of a 3D hierarchical porous solar evaporator by optimizing the ratio of a large-sized porous microstructure through 3D printing (Fig. 2C) [71]. The high salt transport flux mechanism of porous photothermal interface and transport channels based on hydrophilic graphene oxide was analyzed by theoretical modeling and practical performance, demonstrating that a large porous microstructure can provide abundant low-resistance salt ion channels, thus conferring the high salt transport flux (4.3 kg m^–2^ h^–1^, 10 wt.%) to solar evaporator. Continuous and efficient water pumping is an essential factor in the long-lasting operation of solar evaporation systems, and the effect of capillary effects should be fully considered in the system design, where the overall capillary flow rate (*q*) can be assessed by:

$$q=\frac{\mathrm{nr}\pi^{3}\sigma \text{cos}\theta }{4\eta H}$$ (9)

where *n*, *r*, *σ*, *θ*, *η*, and *H* represent the number of capillary tubes, the equivalent radius, the surface tension between the liquid and gas (N m^−1^), the solid–liquid contact angle, the viscosity of the liquid (mm^2^ S^−1^), and the height of the capillary tube (m). Decreasing the equivalent radius (*r*) can increase the capillary effect and reduce the capillary flow rate *q*, while an excessively large *r* can lead to a loss of capillary effect in the evaporator. Therefore, it is reasonable to optimize the construction of a capillary structure to achieve the requirement of excellent anti-salt deposition effect. Considering the above relationships, the anti-salt deposition effect of the evaporator with vertically aligned channel structure of different diameters fabricated by 3D printing was explored by Zhou et al. (Fig. 2D). The results demonstrated that the strategy of salt transfer from the evaporation interface to the bulk water along the shortest path (vertical channels) greatly improved the diffusion and convection efficiency, and achieved long-term desalination [72]. In addition, Koh et al. [73] analyzed the structure and water transport mechanisms of the cellulose bilayer structured solar evaporator fabricated by 3D printing, revealing that the generation of weak cellulose–water hydrogen bonds was the main reason for the decrease in the enthalpy of water vaporization (Fig. 2E).

### Bionic technology

**Fig. 3.**
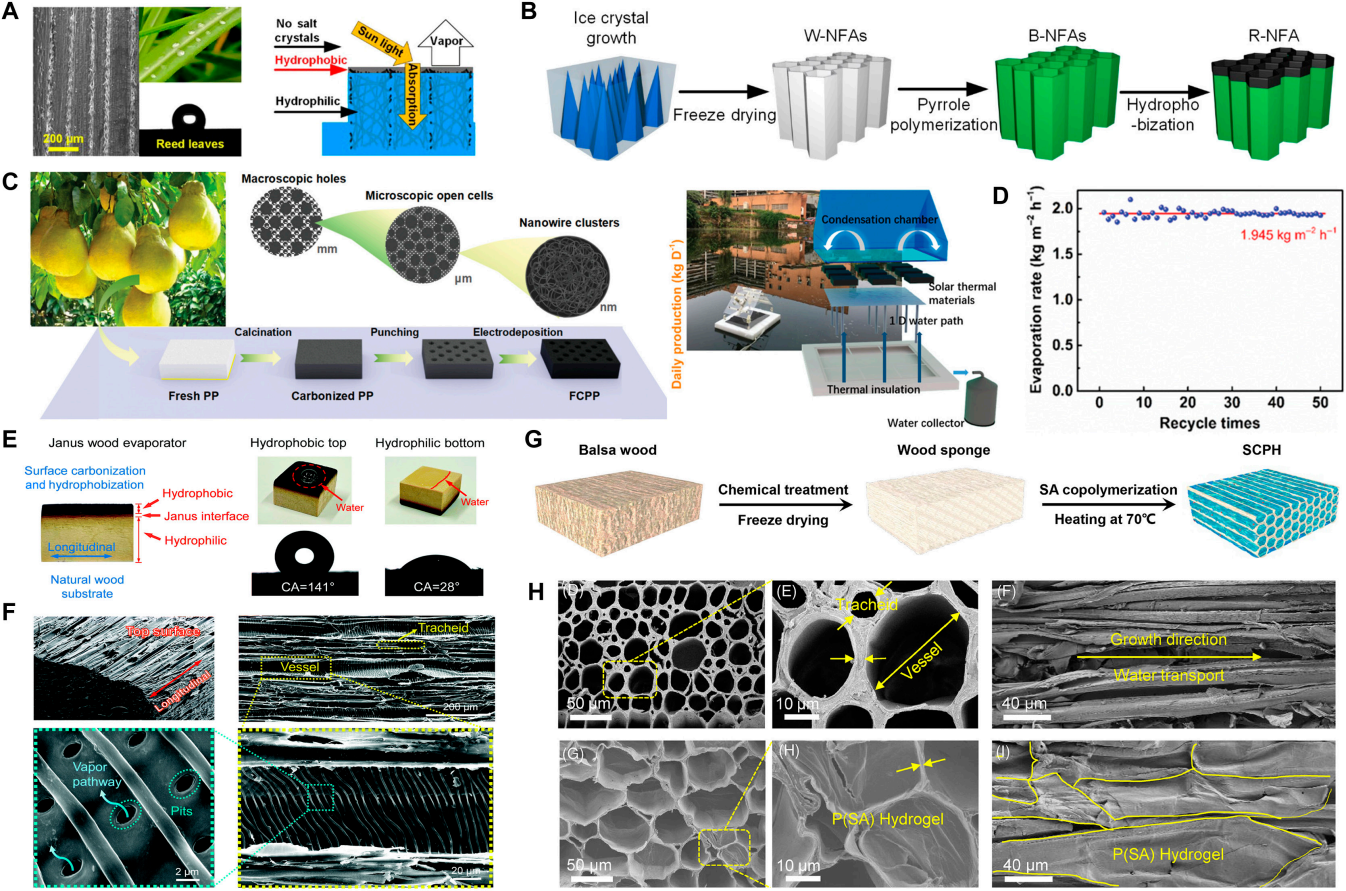
Solar desalination system based on bionic technology. (A and B) The light absorption, water transport, vapor generation, and salt resistance of the reed leaves inspired nanofiber aerogels and schematic diagram of the fabrication process. Reproduced from [74] with permission from the American Chemical Society. (C and D) Schematic illustration of the synthesis route of FCPP and the durability test for 50 cycles. Reproduced from [75] with permission from Wiley-VCH. (E and F) The digital image of a Janus wood evaporator and the contact angle (CA) and rejected water penetration. The SEM image showing the hierarchical structure of the Janus wood. Reproduced from [76] with permission from the Royal Society of Chemistry. (G and H) The preparation and characterization of skeleton constructed polyelectrolyte hydrogel (SCPH). Reproduced from [77] with permission from Wiley-VCH.

Organisms in nature have evolved with appropriate structures to achieve complex biological functions. When designing the structure and function of desalination materials, researchers often build a series of high-performance bionic evaporators inspired by corresponding structures, such as the hydrophobic effect on the surface of lotus leaves and the water transport through plant fiber pores. Inspired by the vascular tissue with transpiration and antifouling functions of reed leaves, Dong et al. designed the bionic hierarchical nanofibrous aerogels with parallel-arranged vessels and hydrophobic surfaces for solar desalination (Fig. 3A and B). The evaporator possessed a parallel-arranged water transport system that can effectively absorb sunlight (~94.8%) and generate steam (1.25 kg m^–2^ h^–1^), similar to the transpiration of a reed leaf, while preventing salt crystallization in the same way that a reed leaf prevents scaling [74]. Interestingly, the bionic fractal structure design strategy was used for efficient desalination. The evaporation enhancement mechanism of fractal structure design was proposed by Yang et al. (Fig. 3C), exploiting the inherent micro-scale porosity of pristine biomass in dehydrated pomelo peel by modifying the structure to obtain a fractal bionic evaporator that allowed the enhanced light capture and energy harvesting with macroscopic pore pattern distribution, showing an evaporation rate of 1.95 kg m^−2^ h^−1^ and excellent desalination (Fig. 3D) [75]. Likewise, multistage pore channels of wood are one of the main objects being referenced, which require surface coating or carbonization to achieve a higher evaporation rate. Among them, CNT, graphene, and metals have been proven as excellent coatings for light absorbers, while salts are dissolved into the native water by diffusion and convection through the internal pores of the wood. Recently, utilizing the Janus absorber (a bilayer structure with opposite properties and different functions), researchers have assigned 2 functions, SVG and water pumping/salt drainage, to different functional layers, while salt is deposited in the hydrophilic layer and dissolves rapidly. This interfacial evaporation effect localizes heat at the liquid–air interface, while the highly open microporous 3D structure facilitates lower thermal conductivity and thus improves solar desalination performance. The thermal conductivity (λ, W m^−1^ K^−1^) of the evaporator can be calculated by

λ=ρ×α×c (10)

where *ρ* is the density (kg m^−3^), *α* represents the thermal diffusivity (m^2^ s^−1^), and *c* is the specific heat capacity (J kg^−1^ K^−1^). Typically, the Janus absorber has a low λ, a thermal characteristic that is ideal for achieving superior performance in solar desalination. Combining these advantages, the unique layered mesoporous structure of the Janus wood evaporator designed by Chen et al. achieved rapid mass transfer and favorable thermal localization, promoted ion diffusion, and facilitated the equilibrium of brine concentration to prevent local salinity increase, achieving an average evaporation rate of >80% as well as an excellent anti-salt effect (Fig. 3E and F) [76]. In addition, Li et al. took the advantage of polyelectrolyte hydrogel and wood sponge evaporation to develop an evaporator with extremely low thermal conductivity (0.109 W m^−1^ K^−1^) and an evaporation rate of 2.13 kg m^−2^ h^−1^ (Fig. 3G and H). By measuring the salt rejection ratio *R* of the evaporator, which was calculated by

$$R=1-\frac{\left(m_{3-}m_{1}\right)\left(1-x\right)}{\left(m_{2-}m_{3}\right)x}$$ (11)

where *m*_1_ is the dry weight of the evaporator, *m*_2_ is the dissolved weight of the evaporator, *m*_3_ is the dried mass of the evaporator after thorough drying after dissolution, and *x* is the concentration of brine, they found that *R* of the evaporator was as high as 88.7%, achieving a record anti-salt effect [77].

### Microfluidic electrospinning technology

**Fig. 4.**
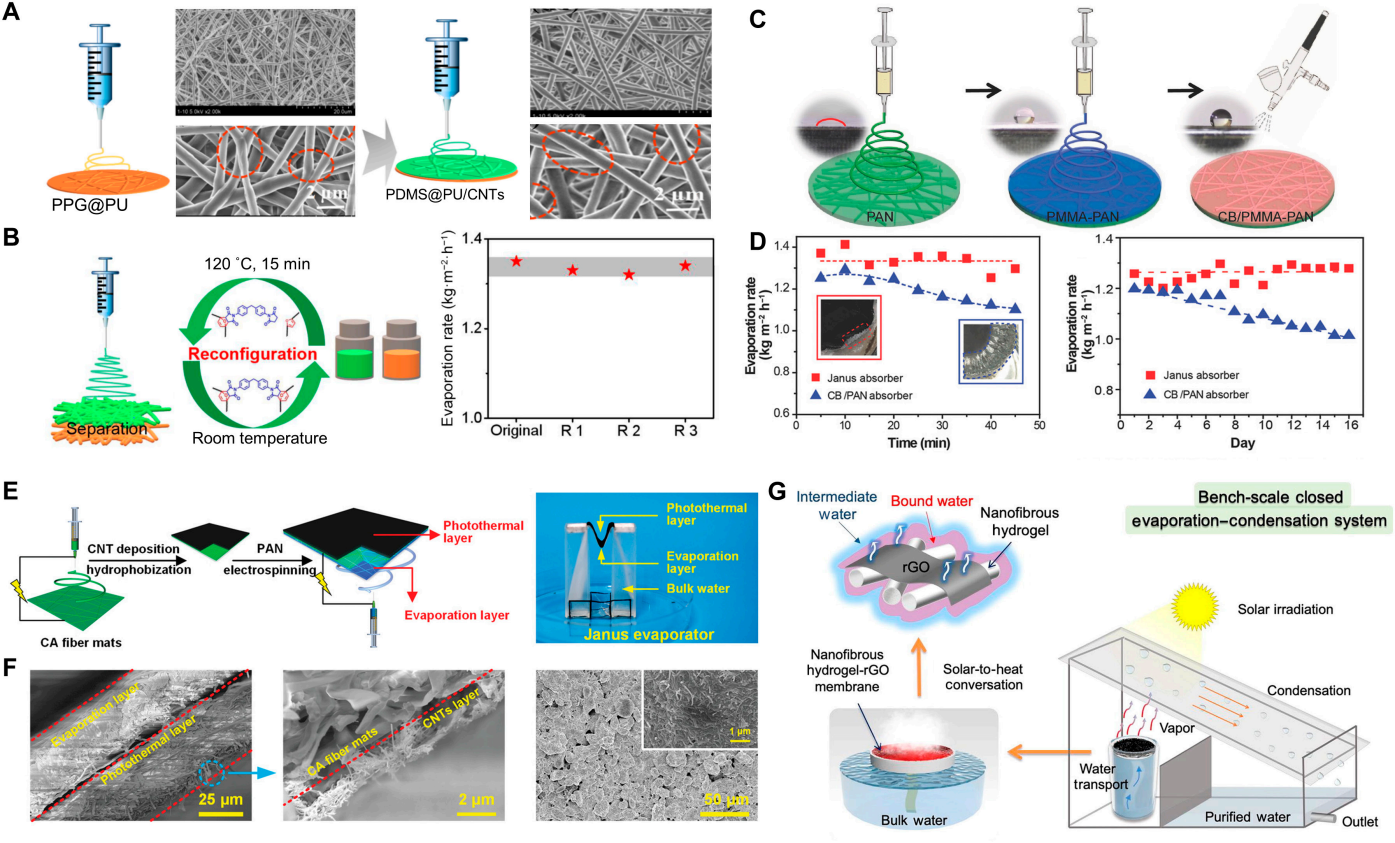
Solar desalination system based on microfluidic electrospinning technology. (A and B) The membrane fabrication by electrospinning, SEM morphology, and schematic diagram of the recovery and reconfiguration process with water evaporation performance. Reproduced from [78] with permission from Springer Nature. (C and D) Flowchart for the fabrication of Janus absorber by electrospinning, and the evaporation rate and the long-term stability of Janus absorber. Reproduced from [80] with permission from Wiley-VCH. (E and F) Schematic of the manufacturing process and hierarchical structure of Janus fibrous mats. Reproduced from [81] with permission from Wiley-VCH. (G) Schematic illustration for the fabrication of the nanofibrous hydrogel-rGO (NHrG) membrane and evaporation device. Reproduced from [82] with permission from Elsevier.

Microfluidic electrospinning technology is an advanced manufacturing method for preparing polymer-based fiber products. Along with the development of nanotechnology and many disciplines, electrostatic spinning plays an essential part in the field of solar desalination as a simple and effective new processing technology that can produce nanofibers. Electrospun nanofiber membranes have high porosity and permeability, which facilitates sunlight irradiation and water transport, and are therefore considered to be an ideal support skeleton for evaporators. A self-repairable evaporator was fabricated by polypropylene glycol-based polyurethane (PPG@PU) and polydimethylsiloxane-based polyurethane-CNTs (PDMS@PU-CNTs) with different wettability by Liu et al. (Fig. 4A), achieving a stable evaporation rate of 1.34 kg m^−2^ h^−1^ (Fig. 4B) [78]. The use of the abundant and highly interconnected pores within the nanofiber membrane can facilitate superior salt resistance and solar evaporation [79]. Xu et al. [80] innovatively combined Janus properties with microfluidic electrospinning and demonstrated that a flexible Janus absorber fabricated by continuous electrostatic spinning could achieve stable and efficient solar desalination (over 16 days), which exhibited high SVG (72%) and stable water output (1.3 kg m^−2^ h^−1^) (Fig. 4C and D). Therefore, the development of Janus membranes using microfluidic electrostatic spinning has become a research hotspot due to their excellent performance in solar irradiation for photothermal conversion, water evaporation, and seawater desalination. The new enhanced modified suspended-type evaporator (STEs) constructed using a Janus fibrous mat had a high evaporation rate of 1.94 kg m^−2^ h^−1^, and due to the reverse wettability of the membrane, the evaporator allowed salt crystallization only on the hydrophilic bottom layer, thus demonstrating zero liquid discharge salt resistance and recovery of minerals from brine (Fig. 4E and F) [81]. Interestingly, the electrostatic spinning nanofiber hydrogel-reduced graphene oxide (NHrG) membrane has been shown to have intermediate water vaporization dominating the desalination process and thus their key role in reducing the vaporization enthalpy can lead to a higher evaporation efficiency (1.85 kg m^−2^ h^−1^) (Fig. 4G). The evaporation enthalpy can be calculated according to the following equation:

$$H=\frac{F}{\frac{\mathrm{dm}}{\mathrm{dt}}}$$ (12)

where *H* is the vaporization enthalpy (J g^−1^), *F* is the heat flow (mW), and $\frac{\mathrm{dm}}{\mathrm{dt}}$ is the mass change as a function of time (mg s^−1^), representing the amount of water being evaporated in a unit of time. As a result, 99.5% salt ion removal was achieved by saturated membrane interfacial evaporation with mainly intermediate water under the key effect of reduced vaporization enthalpy [82]. Desalination devices based on microfluidic electrostatic spinning technology have been shown to have excellent evaporation efficiency and salt resistance, while an in-depth understanding of the theory of controlled interfacial evaporation is still lacking. Meanwhile, desalination systems based on microfluidic electrostatic spinning have been combined with pollution control and interfacial catalysis to achieve multifunctional marine resource utilization effects, which will be discussed in detail in the next chapter.

### Other advanced manufacturing technologies

Evaporators fabricated based on the above advanced manufacturing technologies have achieved excellent evaporation efficiency and salt resistance in a broad sense. Utilizing the thermal localization effect of interfacial evaporation, light absorption and water evaporation are coupled on surface layer, reducing salt accumulation and even directional precipitation [83,84]. Similarly, aerogel-based solar desalination has a corresponding effect. By combining electrostatic spinning and fiber freeze-forming techniques, an elastic ceramic-based nanofibrous aerogel with a cellular architecture possessed high absorbance of 98% and superior evaporation performance (1.50 kg m^−2^ h^−1^). The aerogel showed salt resistance in 20% saline water and under 6-sun irradiation, without any salt crystals on the surface [85]. The porous fluorescent aerogel (CPC aerogel) constructed by Li et al. exhibited an evaporation efficiency of 1.31 kg m^−2^ h^−1^ while enabling the rapid detection and removal of Cr (VI) from the simulated tannery wastewater. The mechanism of desalination and Cr (VI) adsorption was demonstrated in detail, which is a successful attempt of desalination coupled with pollutant removal function [86]. Interestingly, Chinese inks were used as photothermal material for wood surface by the impregnation method (HC-Wood). According to Jurin’s law, capillary action of the HC-wood evaporation process is given by the equation:

$$h=\frac{2\sigma \text{cos}\theta }{\rho \mathrm{gr}}$$ (13)

where *h* is the rising height of salt water in channels, *σ* is the liquid surface tension, *θ* represents the contact angle, *ρ* is the seawater density, *g* is the gravity density, and *r* is the radius of the channel. Thus, motivated by concentration gradient, an autonomous salt exchange occurred between the wood channels through interconnected pits, resulting in dilution of the salt concentration in the wood channels, thus obtaining an evaporation efficiency of 1.6 kg m^−2^ h^−1^ and excellent salt resistance [87]. So far, the development of sustainable evaporation systems with self-salt discharge and water pumping functions remain challenging, and special macro-structural designs facilitate overcoming this limitation. For illustration, using special vertebral structures as solar evaporator, the cylinder can create transport channels between the high and low salinity areas of the evaporator as salt tends to be accumulated on the upper edge of the cone. Thus, the cylinder builds a transport bridge between the high and low salinity regions of the evaporator. Efficient self-salt can discharge and enhance self-water-pumping to the evaporator surface, driven by the salinity difference, resulting in an evaporation rate of 2.8 kg m^−2^ h^−1^ and an extra-long salt-resistance effect (1 month) [88]. Conversely, the generation of oriented salt crystals by structural design is also an option. Huang et al. [89] used the uneven temperature gradient created by light in a 3D conical evaporator to induce salt crystallization at the tip of the vertebrae, achieving an evaporation efficiency of 2.94 kg m^−2^ h^−1^ while enabling the evaporator to collect salt. Unlikely, the novel aerogel design can also facilitate micron-scale brine transport and salt-directed crystallization. As in the case of the PVA hydrogel-based 3D evaporator, which exhibited an enhanced brine transport through capillary pumping and hydrogel expansion, the radial transport of brine in the center can lead to preferential salt crystallization at the edges. As a result, the evaporator achieved an evaporation rate of 2.07 kg m^−2^ h^−1^ and an excellent salt collection capacity [90].

## Current Energy Management Strategies for Solar Desalination Systems

### Phase change materials coupling with the desalination system

**Fig. 5.**
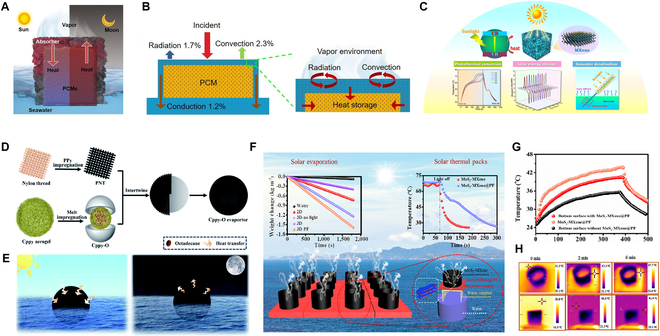
Phase change materials coupling with the desalination system. (A and B) Schematic of the solar-driven PCM-integrated interfacial evaporation and the heat transfer diagram in PCM-integrated architecture during the evaporation process. Reproduced from [91] with permission from Elsevier. (C) Polyimide/MXene hybrid aerogel-based phase change composites for solar-driven seawater desalination. Reproduced from [92] with permission from Elsevier. (D and E) The preparation procedure of the evaporator and schematic diagram of the daytime and nighttime modes for all-day steam generation. Reproduced from [93] with permission from the Royal Society of Chemistry. (F to H) Excellent energy capture of the hierarchical MoS_2_ nanosheets coupled with MXene for efficient solar evaporation and thermal pack. The temperature of the MoS_2_-MXene@PF layer, the bottom surface with the MoS_2_-MXene@PF layer, and the bottom surface without the MoS_2_-MXene@PF layer and the IR photos of 3D-PF under one sun. Reproduced from [95] with permission from Elsevier.

Since solar irradiation is intermittent, heat storage systems have recently been incorporated into desalination systems to allow for all-weather operation. Because the productivity of clean water depends on solar energy utilization or storage of heat, phase change material (PCM)-based energy storage systems can be applied for solar evaporation. A solar-driven PCM-integrated interfacial evaporation system (SPIIE) was demonstrated by Gong et al. (Fig. 5A) [91]. SPIIE could efficiently capture solar energy within its abundant nano/microchannels, and the generated thermal energy was immediately supplied to heat surrounding confined water while transferring the waste heat to the PCM (Fig. 5B). SPIIE with excellent long-term salt resistance possessed a clean water flux of 0.70 kg m^−2^ h^−1^, which was 2.5 times higher than the existing interfacial evaporation systems. Considering the disadvantages of the intermittent SVG process, organic PCMs have also been tried as energy storage systems to generate solar vapor continuously throughout the day. New PCMs based on polyimide (PI)/MXene hybrid aerogels and polyethylene glycol have been used for solar desalination (Fig. 5C). The system showed a high evaporation rate of 1.24 kg m^−2^ h^−1^ and had long-term stability under single-day light [92]. Similarly, the evaporator based on the combination of PCM octadecane/carbonized ultralong PPy nanotube aerogel and the photothermal conversion layer of polypyrrole impregnated nylon thread had a high solar absorption (96%), with an all-weather evaporation efficiency of ~13.4 kg m^−2^ due to the presence of internal PCM (Fig. 5D and E) [93]. Ho et al. [94] conducted a performance evaluation of desalination efficiency and productivity based on the solar evaporator incorporating Fresnel lens and PCM, showing that the energy storage process of the PCM significantly improved the productivity. Further, the performance of the paraffin-based MoS_2_-MXene@PF 3D evaporator can prolong the evaporation process due to the heat release during the phase transition in the case of stable evaporation (Fig. 5F to H). According to the Stefan–Boltzmann law:

$$\Phi =\varepsilon \sigma \left(T_{e}^{4}-T_{0}^{4}\right)$$ (14)

where *Φ* is the heat flux, and the obtained radiant heat and the radiant heat loss for this evaporator are calculated by Guo et al. According to Newton’s law of cooling:

$$Q=h\left(T_{e}-T_{0}\right)$$ (15)

where *Q* represents the heat energy, and the calculated values for both convection heat loss and radiation heat obtained are ~25 W m^−2^. This proved that the photothermal evaporator incorporating PCM possessed excellent energy use efficiency and could be promising for use as thermal preservation equipment [95]. In general, compensating for the mismatch between peak energy supply and peak energy demand and improving efficiency and/or reducing costs by recovering heat originally wasted make energy storage systems an attractive option for solar desalination, but more rigorous efficiency assessments still need to be further explored to meet actual applications.

### Seawater desalination-based multi-coupling system

**Fig. 6.**
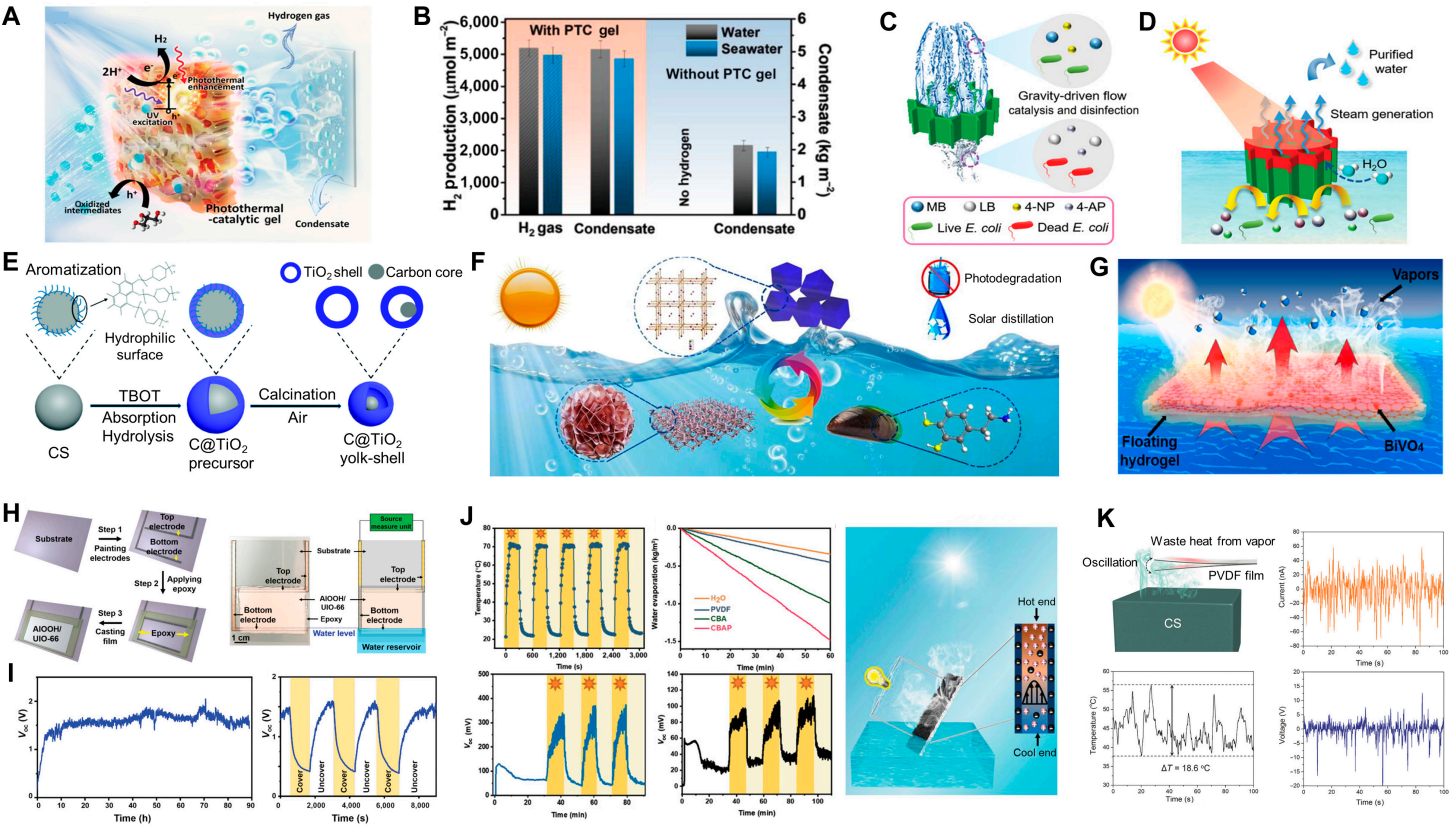
Seawater desalination-based multi-coupling system. (A and B) Schematic drawing of the designed gel for concurrent solar vaporization/hydrogen generation and the amount of hydrogen produced and condensate collected from the HCS without the PTC gel under Xe lamp irradiation. Reproduced from [96] with permission from Wiley-VCH. (C and D) Diagrams showing multiple functions of the biomimetic CS/HAP@Pd aerogel in the gravity-driven continuous flow catalysis/water disinfection and the illustration of solar energy-driven steam generation and water purification. Reproduced from [98] with permission from Wiley-VCH. (E) Schematic illustration of the synthesis steps for evaporation and photothermal–photocatalytic degradation of organic pollutants. Reproduced from [99] with permission from the Royal Society of Chemistry. (F) Mussel-inspired photothermal synergetic system for clean water production using full-spectrum solar energy. Reproduced from [100] with permission from Elsevier. (G) BiVO_4_ and reduced graphene oxide composite hydrogels for solar-driven steam generation and decontamination of polluted water. Reproduced from [101] with permission from Elsevier. (H and I) Schematic illustration of the evaporation device fabrication process and the plot of time-dependent and periodic variations *V*_oc_. Reproduced from [102] with permission from Wiley-VCH. (J) Polyaniline-coated MOF nanorod arrays for efficient evaporation-driven electricity generation and solar steam desalination. Reproduced from [103] with permission from Wiley-VCH. (K) Schematic diagram of the steam generation-induced electric potential. Reproduced from [104] with permission from Wiley-VCH.

The combination of catalysis system with solar desalination deserves more attention recently. There is evidence that this combination has the potential to simultaneously perform solar desalination and water purification or hydrogen production, thereby increasing solar energy utilization and/or reducing contaminant accumulation. Gao et al. designed a H_2_O–H_2_ cogeneration system (HCS) for simultaneous desalination and hydrogen production via a photothermal catalytic (PTC) gel with a hydrophobic membrane for H_2_O/H_2_ co-production, achieving a solar evaporation rate of ~1.49 kg m^−2^ h^−1^ and a hydrogen generation rate of ~3,260 μmol m^−2^ h^−1^ (Fig. 6A and B) [96]. Further evidence indicates that the catalytic-desalination device exhibited a biphasic vapor/catalyst/hydrogen interface, significantly reducing the interfacial potential barrier, which is favorable for improving its practical applicability [97]. Likewise, multifunctional desalination systems with catalytic activity for scalable and sustainable water remediation proved to be feasible. Biomimetic aerogel with solar desalination and wastewater purification functions designed by Xiong et al. [98] possessed a high removal efficiency (97.6%) and evaporation efficiency (86.7%) to efficiently produce clean water from the simulated wastewater and the actual seawater (Fig. 6C and D). Solar evaporators with a photocatalytic capability allow for rapid degradation of aquatic contaminants through surface catalytic effects and therefore possess the potential to purify polluted water and produce clean water immediately. For example, the catalytic-desalination systems based on TiO_2_ (Fig. 6E) [99], CuO (Fig. 6F) [100], BiVO_4_ (Fig. 6G) [101], and other catalysts exhibited efficient photocatalytic oxidation of organic pollutants in the contaminated water, demonstrating the advantages of multi-effective use of light energy.

Recent studies have shown that the process of water evaporation is a promising driver for generating streaming voltage, and this steam-driven power generation offers a new idea for energy supply and a sustainable path to efficient solar energy utilization. Ma et al. [102] designed a 2D AlOOH/UIO-66 (MOFs) hybrid nanomaterial, due to the synergy between the morphology and the high surface potential, which could generate electricity from solar evaporation with an average open circuit voltage (*V*_oc_) of ~1.63 V (Fig. 6H and I). To demonstrate the rational coupling of solar desalination and power generation under AM1.5G, a desalination-power generation system based on a polyaniline-coated MOF nanorod array membrane was synthesized by Li et al. (Fig. 6J). The system continuously generated a sustainable *V*_oc_ of 709.3 mV and a maximum output power density (*P*_max_) of ~15.377 mW m^−2^ at a high evaporation efficiency of 1.866 kg m^−2^ h^−1^, which can efficiently extract energy from the evaporation process while generating fresh water and electricity [103]. Likewise, scavenging the dynamic mechanical and temperature fluctuations of solar steam for waste energy to electricity conversion was proposed by Zhu et al. The principle was to utilize steam generated by solar desalination to excite the thermoelectric and piezoelectric effects of ferroelectric fluoropolymer polyvinylidene fluoride (PVDF), resulting in heating–cooling and slight oscillation of PVDF to generate electricity (Fig. 6K). This thermomechanical response produced a *P*_max_ of 240.7 μW m^−2^, providing an alternative strategy for harvesting waste energy from solar vaporization for power generation [104]. Solar desalination and evaporation-driven power generation may yield interesting combinations, yet for a clear elucidation of the mechanisms involved in various processes such as solar-to-steam conversion as well as electricity, experiments and calculations should be performed to determine the main advantages and disadvantages of the hybrid systems, which may lead to a better understanding and rapid practical applications.

### AI-based model construction and prediction

To address the technical aspects of desalination systems, numerous studies have been devoted to the development of smart multifunctional materials and systems, while little has been done on models for solar desalination and on data frameworks [105,106]. Physical design (e.g., material parameters, structural design, and specific heat exchange area) and system performance are essential for solar desalination systems to be practically applicable; nevertheless, parameters such as ambient temperature, relative humidity, wind speed, solar radiation, inlet water temperature, and total dissolved solids in water for system applications should not be neglected.

**Fig. 7.**
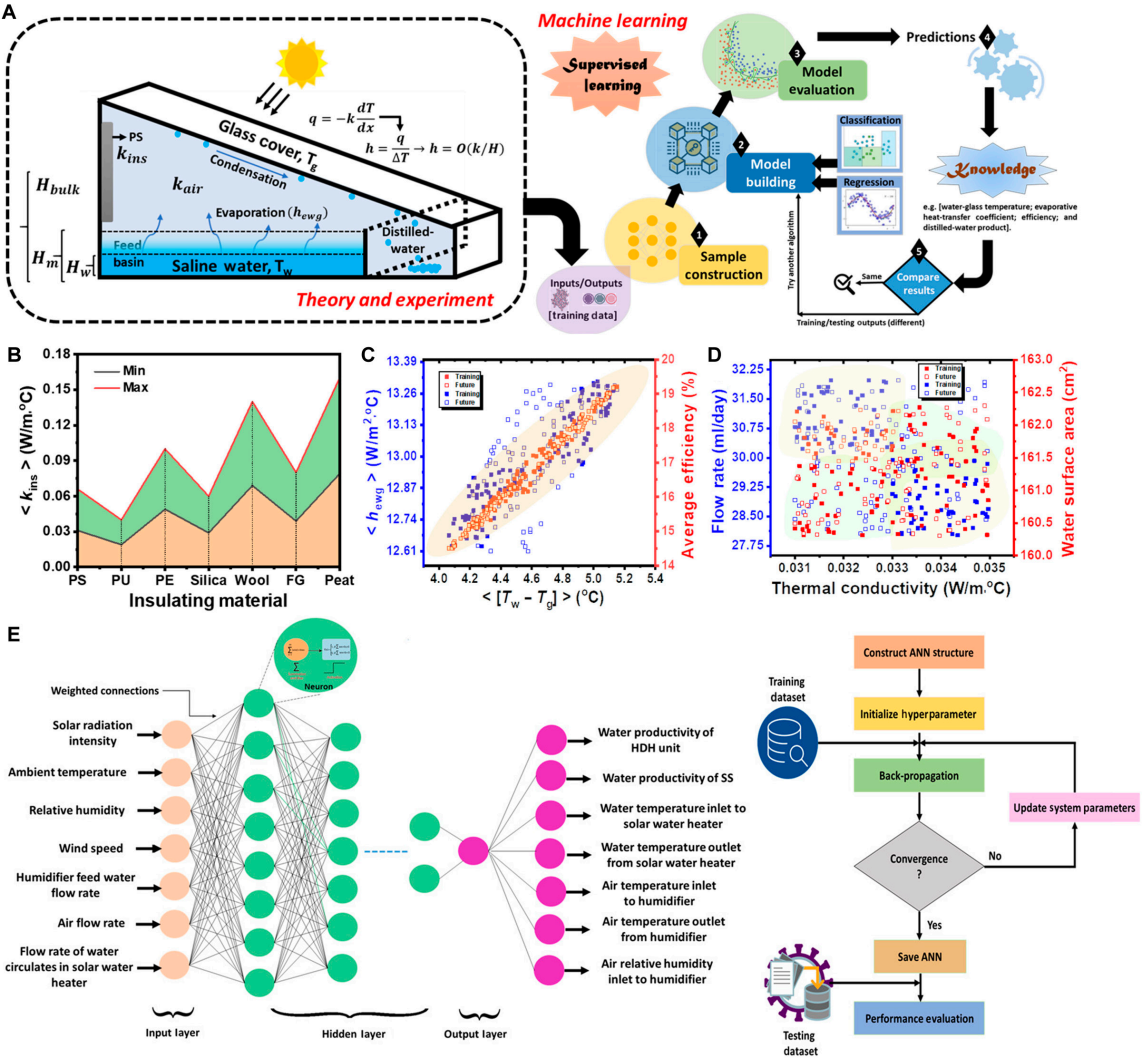
AI-based model construction and prediction. (A) Schematic showing the heat transfer mechanism and other system parameters utilized in the ML analysis, and theoretical modeling, theory, and experiment results are taken as inputs for building training datasets from the PS-based solar still, with the ML algorithm using “stepwise linear regression = (SLR) learner” for prediction of still performance when using different insulating materials. (B) Thermal conductivities of the various studied insulating materials. (C) Output datasets determined for PS training/future relating water-glass temperature difference to both water evaporative coefficient and still efficiency from the input datasets. (D) Input datasets for the PS training/future model calculations. Reproduced from [107] with permission from Elsevier. (E) ANN model used for the SS-HDH system performance prediction and Adam optimizer flowchart. Reproduced from [108] with permission from Elsevier.

AI is an emerging computer science technology that enables industry and research in many fields, including for solar desalination. Supervised ML regression is used to generate trained models from experimental results, and numerous proposed methods aim to develop accurate predictive models through dimensional analysis and intermediate randomized dataset extensions. An important concept in applied AI is to train an agent to find the optimal strategy by actively interacting with the environment in order to maximize expected future gains and achieve goals. With only minor modifications, the corresponding AI models have the potential to be directly extended to other areas. Therefore, optimal material structure and module parameters as well as system configurations can be effectively discovered and applied with the assistance of trained AI predictors. Maddah et al. [107] used the heat transfer theoretical models and experimental results to evaluate the performance of evaporator at different flow rates (Fig. 7A), and the ML tools of Stepwise Linear Regression (SLR) and dataset extensions from between randomization were applied to create training models from the experimental results and randomly generated input datasets, correlating with the outputs through dimensional analysis, allowing them to accurately estimate the theoretical efficiency (or productivity) of the proposed solar evaporator (Fig. 7B and D). In addition, the application of regression algorithms such as multilayer perceptron (MLP), decision trees, and Bayesian ridge regression could achieve good prediction of the productivity of the designed desalination system in the test environment. With the Adaptive Moment Estimation (Adam) optimizer, Salem et al. used an AI regression model to predict desalination efficiency dependent on solar energy with the main objective of obtaining optimal parameters for the MLP (Fig. 7E), including activation function, learning rate, number of hidden layers, number of neurons per layer, and epochs. Their optimized MLP (OMLP) of the trained model has been validated based on statistical measurements, goodness of fit, and error rate, whose OMLP has been compared to the performance of other intelligence-based models with significant improvements [108]. Likewise, Wang et al. proposed a data-driven AI framework for discovering the most efficient graphene nanopores for seawater desalination. Through a combination of deep reinforcement learning (DRL) and convolutional neural networks (CNNs), 7,999 different nanopores were created and evaluated for seawater desalination performance during a 1-week DRL training period, which demonstrated the ability to discover the best graphene nanopores for seawater desalination. With a trained ML property predictor, DRL can automatically learn to effectively and efficiently discover the best material structure to help predict the expected productivity of desalination structures [109]. Numerous examples demonstrate the transformative potential of AI to provide efficiency and cost optimization for the desalination systems, and to bring about the corresponding changes in process performance issues.

## Outlook

The salt resistance of solar desalination has been widely improved by rational design of surface microstructure and internal water supply channels, but most of the current salt resistance tests are still based on laboratory scale, and its actual salt resistance still needs to be obtained in real environment. Most of the evaporators based on the advanced manufacturing technologies mentioned above have taken into account (a) reasonable regulation of the *A_p_*/*A_e_* ratio and the design of a structured self-driven dissolved salt strategy utilizing salinity gradient to quickly transport salt to bulk seawater, (b) leveraging evaporator wettability switching interface to isolate salt from the photothermal structure, and (c) through a subtle structural design, inducing salt to be precipitated in specific areas of the evaporator in a targeted manner [110]. Nevertheless, the corresponding structural design to maintain the salt resistance and evaporation performance still needs further validation. Ongoing work needs to fully elucidate the interactions between seawater and functional materials/structures as well as their impact on water evaporation. The focus for large-scale applications is on developing practical seawater purification and storage systems while addressing the durability of evaporators in seawater and even contaminated water sources.

Based on improving and enhancing the performance of a desalination system, AI techniques are advocated to obtain good modeling and testing through experimental datasets without changing the system structure [105,111]. Although methods such as AI and thermodynamic models have been developed to study the evaporation potential of the proposed system, more consideration is given to solar irradiance, ambient temperature, water temperature difference, evaporation temperature difference, and evaporation and condensation temperatures as independent parameter input in the setup. Considering the need to create more AI models in future developments, both early and future experiments should be used to construct such models that include design modifications, insulation, operating/weather conditions, and the effect of nanofluids on static performance. Meanwhile, a comprehensive technical and economic sustainability analysis should be performed by AI to determine the best evaporator preparation route for cost-effective desalination.

By designing the structure of the evaporator unit, fast and massive evaporation performance can be achieved using the assistance of the surrounding environment. The incorporation of PCMs enables evaporators the opportunity to efficiently utilize latent heat of evaporation to quickly address the above bottlenecks and maximize the production of clean water, which requires evaporators to efficiently capture solar energy and converting it into heat energy, ensuring efficient fluid transfer while heat is immediately available for local heating and vaporization of the interface water, and waste heat is transferred and stored in the PCMs, thus greatly reducing heat dissipation. The development of new PCMs should meet the ideal criteria of effectively combining heat storage and interfacial evaporation, and PCMs that undergo phase change processes in a relatively narrow temperature range and are capable of storing and releasing large amounts of heat should be selected for energy storage applications in solar desalination.

The exploitation of the abundant energy in the ocean based on solar desalination deserves to be promoted [112]. Concentration gradients, pressure gradients, and temperature gradients can be applied to thermal osmosis energy conversion such as salt differential power generation, piezoelectricity, and thermoelectricity [113–116]. Reverse electrodialysis (RED) technology consists of multiple sets of cation-selective and anion-selective membranes used alternately between seawater and surface water, which results in a superimposed electrochemical potential difference between the 2 electrodes. The combination of solar desalination with RED technology highlights the revolution that desalination technology can bring to emerging energy production [117]. For thermoelectric technologies that use low-level heat sources (<100 °C) generated during the desalination process to provide energy, current researches had focused on electricity production using the heat difference between the steam or thermal evaporation interface and the environment, and more low-level heat sources need to be found and exploited. In addition, solar desalination can be combined with reverse osmosis technology, where high salinity seawater can be used as a feedstock for power generation, or the salinity gradient energy of seawater can be extracted first, and the drainage water with reduced salinity can be supplied to the reverse osmosis system, a model that has shown some potential for development. However, the challenges facing these coupled technologies are poor cyclability, susceptibility, and negative environmental effects. Therefore, a comprehensive technical and economic/environmental feasibility analysis of the development and design strategies should be conducted to meet the practical requirements [118,119].

## Conclusion

To bring solar interfacial desalination systems rapidly closer to practical use, this review discussed the thermal conversion, energy flow, and salt deposition mechanisms of solar-driven desalination systems based on advanced manufacturing technologies, and determines their feasibility for coupling with multi-stage energy utilization systems and emerging AI technologies, while more research is still needed to elucidate the interactions between seawater and functional materials/structures (latent heat recovery, heat loss minimization, and practical configurations). Considering the feasibility of building a large-scale integrated desalination operation system using solar evaporators as the original components, a comprehensive evaluation of parameters such as cost optimization, desalination efficiency, and energy utilization is required, leading to the development of a low-cost and highly efficient integrated seawater treatment system. Integrated systems combining water collection, purification, transportation, and wastewater reuse need to be developed for practical solar-driven clean water production. Addressing the above issues will help accelerate the practical application of solar-driven solar desalination systems and provide a referable pathway to solve the world’s current freshwater/energy crisis.
